# Energy expenditure of acutely ill hospitalised patients

**DOI:** 10.1186/1475-2891-5-9

**Published:** 2006-03-29

**Authors:** Salah Gariballa, Sarah Forster

**Affiliations:** 1 Sheffield Institute for Nutritional Studies on Ageing, The University of Sheffield, Northern General Hospital, Sheffield, S5 7AU, UK; 2 Human Nutrition Unit, The University of Sheffied, Sheffield, S5 7AU, UK; 3 Department of Internal Medicine, Faculty of Medicine and Health Sciences, PO Box 17666, Al Ain, United Arab Emirates University

## Abstract

**Objective:**

To measure energy expenditure of acutely ill elderly patients in hospital and following discharge in the community.

**Design:**

Sixty-three consecutive hospitalised acutely ill elderly patients were recruited. Eight patients were studied to assess the reliability of the Delta Tract Machine as a measure of energy expenditure; 35 patients had their energy expenditure studied in hospital on two occasions and 20 patients had their energy expenditure measured in hospital and at 6 weeks in the community

**Results:**

Men had higher basal energy expenditure (BMR) values compared to women however the difference was not statistically significant [Men, mean (SD) 1405 (321) Kcal, women 1238 (322) kcal; mean difference (95% CI) 166 kcal (-17 to 531), p = 0.075]. After adjusting for age, gender and body mass index both medication and C-reactive protein (CRP), concentrations showed significant correlation with measured energy expenditure in hospital, (r = -0.36, "p < 0.05"; r = -0.29, "p < 0.05" respectively). However, in a multivariate analysis for all 63 subjects combined CRP explained most of the variance in BMR in hospital. The Harris Benedict equation predicted within ± 10% measured BMR in only 47% of individuals in hospital.

**Conclusion:**

Tissue inflammation and medications were associated with change in measured energy expenditure in acutely ill patients.

## Introduction

Increasing numbers of older people worldwide has created a need for additional knowledge of age-related changes relevant to nutrition, which has importance in the treatment and prevention of disease and in maintaining good health and quality of life in an ageing population [1,2]. Ill health frequently has an adverse effect on nutritional status of older people. For most, these effects are limited to the time of acute illness and the temporary nutritional disadvantage is overcome depending on the body reserve once the customary pattern of eating is resumed [2]. But if episodes of ill health occur repeatedly or become prolonged, as is the case in older people nutritional status may decline progressively [2-4].

Assessing the energy needs of acutely ill elderly patients is vitally important in order to prevent energy and nutrient deficiencies. To date the energy requirements of elderly patients are not clear due to difficulties of measurement of energy intake from self recorded diaries and/or dietary interviews [2]. Measurement of energy expenditure in elderly patients is one important way of establishing estimates of energy requirements in both health and disease [5]. However, there is a lack of relevant studies and most prediction equations used to calculate energy expenditure in acutely ill elderly patients are based on young individuals [2]. Furthermore presence of chronic diseases, disabilities, drug intake and heterogeneity of physical activity and body composition make it difficult to obtain accurate data on energy expenditure in the elderly population [5,6].

The aim of this study was therefore to measure the energy expenditure of acutely ill elderly patient in hospital and following discharge in the community.

## Subjects & methods

### Patients

The study was conducted over an 8 months period at a 650-bed Associate Teaching Hospital in South Yorkshire. The integrated medical unit has 168 beds on 7 wards admitting unselected patients on the basis of need. Sixty-three hospitalised acutely ill elderly patients were recruited. Eight patients were recruited to assess the reliability of the Delta Tract Machine for measuring energy expenditure in acutely ill elderly patients; 35 patients had their energy expenditure studied during their hospital stay on two occasions; 20 patients had their energy expenditure measured both in hospital and 6 weeks later in the community. Patients suffering from severe medical or psychiatric illness, morbid obesity, moderate to severe dementia or difficulty with swallowing were excluded. The Local Health Ethical Committee approved the study and all patients or their carers gave written informed consent. All patients had demographic and medical data collected including current diagnosis, history of chronic illnesses, smoking, alcohol and drug intake

### Measurements of basal metabolic rate (BMR) [indirect calorimetry] [7]

Basal metabolic rate (BMR) which reflects the energy requirements for maintenance of the intracellular environment and the mechanical processes such as respiration and cardiac function was measured using the Delta tract machine (Helsinki, Finland). This method of indirect calorimetry uses a plastic ventilated hood, which delivers a known quantity of gases to the subject, and a sample line which feeds back to the main machine, this then measures the amount of oxygen and carbon dioxide in the expired air; and calculates respiratory quotient, and energy expenditure. Reliability was ensured by calibrating the machine immediately before each measurement using a reference gas mixture comprised of 5% carbon dioxide and 95% oxygen. Each individual measurement lasted for 40 minutes and was taken in the morning, around 8.00 am with the patient lying in the bed and after overnight fast (from midnight only water was allowed).

### Measurements of Resting Metabolic Rate (RMR)

Thermogenesis encompasses a wide variety of phenomena which include energy expenditure and heat generation associated with feeding, body temperature maintenance and thermogenic response to various specific stimuli such as smoking, caffeine, and drugs. Over the course of the day this builds up and is currently thought to account for about 10% of total energy expenditure. In order to take account of the thermic effect of diet on the energy expenditure of the non-fasting sedentary hospitalized subjects, ten percent of measured BMR was added for dietary induced thermo genesis.

### Measurements of Total Energy Expenditure (TEE)

Physical activity accounts for 20–40 % of total daily expenditure in most individuals. However there is a wide variation in the energy cost of any activity both within and between individuals. For subjects in the community the BMR measurement was multiplied by 1.3 to take account of physical activity plus 10% of BMR for the thermic effect of food.

#### Quantification of the Delta Tract Machine measurement error

We performed a set of replicate readings, obtained by measuring BMR of 8 elderly patients (5 male), of mean age (SD) 75 years [8], on two consecutive days after adjusting for meal times (after 4 hours), medications and severity of illness (CRP).

As the difference between the observed value, with measurement error, and the subject's true value will be at most two standard deviations or within twice the coefficient of variation with probability 0.95. The precision for the BMR data for this study were as follows: BMR within two standard deviations (BMR 2 × 113 = 226 kcal, or within twice the coefficient of variation (BMR 2 × 5.9 % = 11.8%).

### Other measurements

Each patient's nutritional status was determined from anthropometric and biochemical data. Weight was measured with a portable mechanical chair scale to 0.1 kg in a hospital gown. Height was inferred from demispan (using the following equation: Men 1.2 × Demispan + 72; Women 1.2 × Demispan + 67) to determine body mass index (BMI) = [weight (kg) ÷ {height (m)}^2^]. Mid-upper arm circumference (MUAC) was measured by a flexible tape measure at the level of the midpoint between acromion and olecranon, with the elbow flexed at 90°. Triceps skin fold thickness (TSF) was taken at the same level over the triceps with the arm hanging relaxed at the side. TSF was measured using Harpenden Skin fold callipers accurate to 0.2 mm (Practical Metrology Sussex UK) and the mean of three measures was recorded. C-reactive protein (CRP) concentration, a marker of acute inflammation (severity of illness) was measured by a modified latex-enhanced immuno-turbidimetric assay (normal range < 6 mg/L). The inter-assay coefficient of variation was 3.9%.

### Data analysis

Repeated measures analysis of variance (ANOVA) test was used to test within and between subject differences and p value < 0.05 was considered significant. Differences between groups at baseline were adjusted for history of drug intake, severity of acute illness (CRP), smoking and alcohol consumption and chronic illness. Mann-u-Whitney test, Partial and Spearman's rank correlation were also used. Forward stepwise multiple regression analysis was performed to determine the predictive importance of baseline variables (age, gender, body weight, body mass index, drugs, and CRP concentration) for measured energy expenditure. Adjusted and change in R^2 ^values are presented.

## Results

Energy expenditure measurements were obtained for 8 patients on two consecutive days following acute admission; for 35 patients within 72 hours of admission and 5 days later in hospital; and for 20 patients in hospital and 6 weeks later following discharge in the community. Underlying diagnoses were ischaemic heart disease (23), stroke (8), falls (6), syncope (4), heart failure (3), cheat infection (2), back pain (1), and miscellaneous (16).

Baseline clinical characteristics, anthropometeric and energy expenditure data for each patient group are presented in table 1.

**Table 1:** Patients baseline characteristics and BMR, RMR and TEE at baseline 5 days and in the community respectively [mean (SD)]

**Variable**	**Baseline (n-55)**	**Day 5 (n = 32)**	**Six weeks in the community (n = 17)**
Age	76 (9)		
Gender, female (%)	8 (40%)		
Chronic disease/patient	1.5		
Drug/patient+	2.1		2.1
Body weight (kg)	70 (13)		72 (12)
BMI (kg/m^2^)	26.3 (5.5)		26.4 (5.5)
MUAC (cm)	29.3 (2.9)		29.7 (1.9)
TSF (mm)	19 (7.8)		16.3 (4.4)
Thyroid stimulating hormone (normal 0.38–4.7 mμ/L)	1.6 (0.75)		3.3 (6.9)
C-reactive proteins * (normal <6 mg/L)	22 (28)	12 (18)	5 (7)
Energy Expenditure [BMR] * (Kcal/day)	1403 (346)	1317 (446)	1159 (445)

* P value for difference between hospital and community values < 0.05 + Diuretics, ACE inhibitors, β-blockers, Calcium channel blockers, statins, aspirin warfarin, clopidogrel, analgesics, benzidiazepines, antidepressants and oral hyopglycaemics

CRP concentration, drugs and male gender showed significant positive correlation with measured energy expenditure in hospital. Men had higher BMR values compared to women however the difference was not statistically significant [Men, mean (SD) 1405 (321) Kcal, women 1238 (322) kcal; mean difference (95% CI) 166 kcal (-17 to 531), p = 0.075]. After adjusting for age, gender, body mass index both Drugs and CRP concentrations showed significant correlation with measured energy expenditure in hospital (r = 0.36, p value < 0.039; r = 0.29, p value < 0.042 respectively). However, in a multivariate analysis for all 63 subjects combined CRPs a measure of tissue inflammation explained most of the variance in baseline measured energy expenditure in hospital.

**Figure 1:**
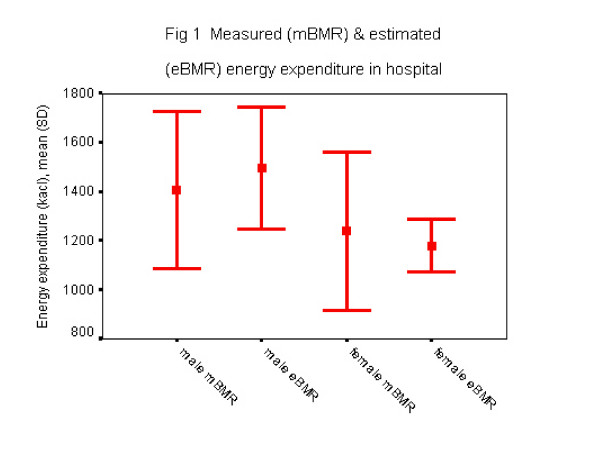
Measured (mBMR) and estimated (eBMR) energy expenditure in hospital

Although calculated energy expenditure using Harris-Benedict equation approximates measured energy expenditure during acute illness (figure 1), the equation predicted within ± 10% measured BMR in only 47% of individuals.

## Discussion

The main findings of this study were that CRP concentration a marker of severity of acute illness and drugs mainly vasoactive ones, aspirin and analgesics showed a significant and independent association with measured energy expenditure in hospital. The Harris Benedict equation predicted within ± 10% measured BMR in only 47% of individuals in hospital.

Studies of elderly people in hospitals are in agreement that food intakes are less than those reported for free-living elderly people [2,8,9]. The reasons for this are likely to be the combined effects of poor food intake as well as the extra energy cost of the metabolic disturbances associated with illness or disability. Measurement of energy expenditure is therefore important in establishing the energy requirements of older people during acute illness. The information obtained will be clinically important in maintaining adequate supply of nutrients in order to replete energy stores and lean tissues especially for those with low nutritional reserves.

The main determinants of energy expenditure in man are body size, body composition, physical activity, age, gender, diet, genetic factors, hormonal and psychological state, pharmacological agents and disease process [10]. With advancing age undernutrition, chronic diseases and drug intake become more common. During acute illness a series of metabolic events are activated that leads to a state of negative nitrogen balance and significant loss of lean body mass. This process is mediated by inflammatory cytokines and is characterised by marked anorexia and net whole body protein breakdown [11]. Basal metabolism may consequently increase by up to 10% for each 1°C rise in body temperature [2,11].

We have found in this study that CRP concentration a marker of severity of acute illness and drugs were associated with a significant increase in energy expenditure. The hypermetabolic state of acute illness and the accompanying neuroendocrine changes strongly influence energy, protein and other nutrients metabolism. For example, gluconeogenesis and glycogenolysis, enhanced lipolytic activity and catabolism of muscle protein are characteristics metabolic responses to catabolic stress associated with acute illness [6]. These changes in metabolism particularly if combined with pre-existing undernutrition which is very common in elderly patients will lead to poor clinical outcome including poor physical function, increased risk of infection and length of hospital stay. Besides the effect of acute illness many drugs commonly used in the elderly may influence energy expenditure such as aspirin, beta-blockers, anti hypertensive and other vasoactive drugs. Drugs can produce either an increase or decrease in BMR. For example, Dempsey et al. [12], have shown that in critically ill patients as the dosage of sedatives increased, energy expenditure decreased with some individuals reaching only 60% of predicted energy expenditure on the high doses of sedatives.

Although we have adjusted for physical activity for community patients (Physical Activity taken as an increase of 30% of RMR), we have not done so for the hospitalised cohort because of the sedentary nature of these patients. We have attempted to measure physical activity in the community using a proxy questionnaire (Yale Physical Activity Survey [YPAS]). However, we abandoned using YPAS scores, because we found the accuracy of physical activity reporting as assessed by this proxy measure to be a problem among patients. Many researchers have found physical activity, the most variable component of daily energy expenditure. It comprises as little as 15% of daily energy expenditure in sedentary older people and up to 50% of daily energy output in highly active older people[10]. Furthermore very few studies to date have measured the accuracy of available methods for measurement of physical activity at all ages but particularly in the elderly. Physical activity still remains an important determinant of energy. Accurate methods for assessment of physical activity in the elderly are therefore needed[10].

Although our aim was to recruit acutely ill patients judging by the CRP concentrations many of our subjects were only minimally stressed. Given the nature of the study very sick patients were less likely to give consent to take part in the study. It is also well known that BMR varies through out the day and also considerably from day to day especially during the early phase of acute illness. Another potential weakness was that many subjects did admit to food and or caffeine consumption 2–4 hours prior to energy expenditure measurement.

Little is known so far about energy expenditure and requirements of acutely ill elderly patients. This group of patients is at risk of negative energy balance, which may lead to undernutrition, because of hypermetabolism associated with acute illness. However, research to date provides no evidence that positive energy balance during critical/acute illness prevent protein-energy undernutrition. It is indeed the case that in clinical practice the timing and the amount of nutritional support in these patients especially those who are critically ill is less clear, because of lack of evidence.

There is however, an urgent need for research, which help the understanding of the metabolic response to tissue inflammation and injury associated with acute illness in elderly patients. This will help provide guidance on optimal timing, route and composition of nutritional therapy relative to a patient's metabolic stress, age and specific illness and therefore improve their outcome.

In conclusion this study showed that acute inflammation and medications during acute illness might affect energy expenditure of hospitalised older people.

Further studies on the impact of acute illness on the timing, macronutrient balance and energy provision of nutritional support of older people is long overdue.

**Table 2:** Multiple regression analysis of age, gender, body weight, drug intake and CRPs with measured resting energy expenditure as the dependent variable.

**Variable/independent predictor**	**R square**	**R square change**	**P value**
	
**(n = 63)**			
Age	.004	.004	.679
Gender	.055	.051	.133
Drugs	.118	.062	.093
Body weight	.124	.006	.594
CRP	.209	.086	.044*

*P value < 0.05
